# Supplementary material for: Changes in gene expression in human skeletal stem cells transduced with constitutively active Gsα correlates with hallmark histopathological changes seen in fibrous dysplastic bone
Source: PLoS One. 2020 Jan 30;15(1):e0227279. doi: 10.1371/journal.pone.0227279 (PMC6991960; doi:10.1371/journal.pone.0227279)
Supplement: S1 Table — FC, fold change. T test, paired t-test applied comparing LV-GsαR201C to mock treated hBMSCs. (DOCX) [file pone.0227279.s001.docx]

**Supporting information**

| **HGNC symbol** | **FC** | **T test** |
| --- | --- | --- |
| ACAN | **-27.11** | 0.053 |
| ITGA8 | **-20.09** | 0.135 |
| RNA5SP392 | **-19.43** | 0.130 |
| VMO1 | **-18.17** | 0.189 |
| LMO7DN | **-11.40** | 0.063 |
| B3GALT2 | **-11.02** | 0.081 |
| HSPB7 | **-10.31** | 0.096 |
| CSPG4 | **-9.97** | 0.086 |
| KRTAP1-5 | **-9.97** | 0.173 |
| FGD4 | **-9.03** | 0.119 |
| CSPG4 | **-8.73** | 0.186 |
| RCAN2 | **-8.73** | 0.018 |
| B3GALT2 | **-8.44** | 0.014 |
| C2orf40 | **-8.17** | 0.029 |
| SLITRK4 | **-7.90** | 0.057 |
| TNS3 | **-7.39** | 0.037 |
| PTGIS | **-6.69** | 0.112 |
| POSTN | **-6.25** | 0.000 |
| CRIP1 | **-5.85** | 0.031 |
| TEK | **-5.85** | 0.153 |
| BMP6 | **-5.47** | 0.195 |
| RABGAP1 | **-5.29** | 0.019 |
| TRPC6 | **-5.12** | 0.008 |
| KCNB1 | **-4.95** | 0.023 |
| RIMS1 | **-4.79** | 0.234 |
| CD9 | **-4.63** | 0.203 |
| LOC646324 | **-4.63** | 0.079 |
| KCNB1 | **-4.48** | 0.080 |
| SLC18B1 | **-4.48** | 0.098 |
| DACT1 | **-4.48** | 0.148 |
| PPFIBP2 | **-4.33** | 0.008 |
| TNFRSF11B | **-4.33** | 0.137 |
| LMO7 | **-4.19** | 0.081 |
| TNS3 | **-4.19** | 0.002 |
| HDAC9 | **-4.19** | 0.068 |
| MYO10 | **-4.19** | 0.136 |
| FNDC1 | **-4.19** | 0.212 |
| ITGA8 | **-4.06** | 0.009 |
| MEF2A | **-4.06** | 0.061 |
| ANKRD44 | **-4.06** | 0.107 |
| RABGAP1 | **-4.06** | 0.063 |
| RAMP1 | **-4.06** | 0.013 |
| PPP1R14C | **-3.92** | 0.155 |
| LIMS2 | **-3.79** | 0.083 |
| MEF2A | **-3.79** | 0.089 |
| STEAP3 | **-3.79** | 0.021 |
| KIAA1671 | **-3.67** | 0.100 |
| FRY | **-3.67** | 0.151 |
| SLC38A4 | **-3.67** | 0.036 |
| DOCK10 | **-3.55** | 0.010 |
| TNFRSF11B | **-3.55** | 0.136 |
| TPM1 | **-3.55** | 0.098 |
| LRRC2 | **-3.55** | 0.095 |
| NAV3 | **-3.55** | 0.138 |
| AHNAK2 | **-3.43** | 0.043 |
| TJP2 | **-3.43** | 0.112 |
| ANKRD44 | **-3.43** | 0.252 |
| TMEM173 | **-3.43** | 0.048 |
| FOXC2 | **-3.32** | 0.133 |
| CCNYL1 | **-3.32** | 0.187 |
| POSTN | **-3.32** | 0.064 |
| TPST2 | **-3.21** | 0.063 |
| BAMBI | **-3.21** | 0.102 |
| CD14 | **-3.21** | 0.198 |
| C15orf52 | **-3.21** | 0.111 |
| HDAC5 | **-3.21** | 0.045 |
| STEAP3 | **-3.11** | 0.058 |
| CAMK2N1 | **-3.11** | 0.099 |
| PHACTR2 | **-3.00** | 0.144 |
| GPC6 | **-3.00** | 0.132 |
| RFTN1 | **-3.00** | 0.082 |
| CAND2 | **-3.00** | 0.063 |
| CRYAB | **-3.00** | 0.042 |
| DKK1 | **-3.00** | 0.258 |
| KLF2 | **-3.00** | 0.053 |
| RNF141 | **-3.00** | 0.221 |
| VEGFC | **-3.00** | 0.042 |
| USP53 | **-2.91** | 0.002 |
| FADS3 | **-2.91** | 0.029 |
| CCNYL1 | **-2.91** | 0.140 |
| RNF144B | **-2.91** | 0.124 |
| C12orf75 | **-2.91** | 0.012 |
| DUSP4 | **-2.91** | 0.113 |
| SPINT2 | **-2.91** | 0.109 |
| CAMK2N1 | **-2.81** | 0.080 |
| DAPK1 | **-2.81** | 0.012 |
| DMPK | **-2.81** | 0.022 |
| EFHD1 | **-2.81** | 0.128 |
| PXN | **-2.81** | 0.225 |
| KCNK6 | **-2.81** | 0.211 |
| MFGE8 | **-2.81** | 0.063 |
| SIRPA | **-2.81** | 0.085 |
| ZFP36L2 | **-2.81** | 0.108 |
| KANK1 | **-2.72** | 0.086 |
| PRKCDBP | **-2.72** | 0.203 |
| BMP2 | **2.72** | 0.023 |
| CFI | **2.72** | 0.014 |
| KCNJ8 | **2.72** | 0.024 |
| ITPRIP | **2.72** | 0.101 |
| KLHL13 | **2.72** | 0.121 |
| PLXNA4 | **2.72** | 0.122 |
| PTGFR | **2.72** | 0.001 |
| PTP4A1 | **2.72** | 0.002 |
| TMEM158 | **2.72** | 0.053 |
| SLC22A3 | **2.72** | 0.025 |
| PTGFR | **2.81** | 0.031 |
| OSMR | **2.81** | 0.109 |
| NAP1L1 | **2.81** | 0.103 |
| BOC | **2.91** | 0.106 |
| WISP1 | **2.91** | 0.139 |
| FAM134B | **2.91** | 0.069 |
| KCNK15 | **2.91** | 0.084 |
| NLGN4X | **2.91** | 0.086 |
| PAPPA | **2.91** | 0.095 |
| PGM2L1 | **2.91** | 0.135 |
| PHC2 | **2.91** | 0.002 |
| PLPP1 | **2.91** | 0.032 |
| PRSS35 | **2.91** | 0.183 |
| STAMBPL1 | **2.91** | 0.118 |
| TFPI | **2.91** | 0.053 |
| TMEM100 | **2.91** | 0.074 |
| FAM65B | **3.00** | 0.042 |
| CYTL1 | **3.00** | 0.080 |
| KLRC2 | **3.00** | 0.031 |
| PITPNC1 | **3.00** | 0.213 |
| WNT5A | **3.00** | 0.055 |
| PAPPA | **3.00** | 0.002 |
| MRVI1 | **3.00** | 0.106 |
| CRISPLD1 | **3.11** | 0.091 |
| GRIA3 | **3.11** | 0.031 |
| ST8SIA4 | **3.11** | 0.152 |
| SULF2 | **3.11** | 0.170 |
| GLCCI1 | **3.11** | 0.117 |
| LPL | **3.11** | 0.011 |
| PLPP1 | **3.11** | 0.044 |
| RAB27B | **3.11** | 0.031 |
| ANTXR1 | **3.21** | 0.081 |
| APOE | **3.21** | 0.009 |
| CHL1 | **3.21** | 0.020 |
| FAM13C | **3.21** | 0.000 |
| KCNK3 | **3.21** | 0.027 |
| LRRC15 | **3.21** | 0.056 |
| SOX4 | **3.21** | 0.055 |
| IFI44L | **3.32** | 0.019 |
| KIAA1644 | **3.32** | 0.086 |
| MMP13 | **3.32** | 0.027 |
| PDE4D | **3.32** | 0.044 |
| PTGS1 | **3.32** | 0.063 |
| RGS2 | **3.32** | 0.007 |
| RORB | **3.32** | 0.239 |
| SLIT3 | **3.32** | 0.014 |
| DIRAS3 | **3.43** | 0.062 |
| FANK1 | **3.43** | 0.169 |
| PAPPA | **3.43** | 0.094 |
| FAM162A | **3.43** | 0.085 |
| CFAP69 | **3.43** | 0.077 |
| HPD | **3.43** | 0.029 |
| IL18R1 | **3.43** | 0.031 |
| SIK2 | **3.43** | 0.052 |
| CD55 | **3.55** | 0.005 |
| CXCL16 | **3.55** | 0.090 |
| NR4A2 | **3.55** | 0.065 |
| SLC5A3 | **3.55** | 0.049 |
| WDR86 | **3.55** | 0.265 |
| TBX3 | **3.55** | 0.099 |
| ENTPD1 | **3.67** | 0.036 |
| FBXO32 | **3.67** | 0.099 |
| S1PR1 | **3.67** | 0.020 |
| GALNT15 | **3.67** | 0.123 |
| LIFR | **3.67** | 0.121 |
| PDGFD | **3.67** | 0.077 |
| PITPNC1 | **3.67** | 0.138 |
| PTGFRN | **3.67** | 0.094 |
| SFRP1 | **3.67** | 0.005 |
| WISP1-OT1 | **3.79** | 0.173 |
| NDNF | **3.79** | 0.079 |
| CLU | **3.79** | 0.032 |
| DPYSL4 | **3.79** | 0.026 |
| PRR5L | **3.79** | 0.077 |
| ID4 | **3.79** | 0.033 |
| MCTP2 | **3.79** | 0.047 |
| SFRP2 | **3.79** | 0.087 |
| S1PR3 | **3.92** | 0.122 |
| OSMR | **3.92** | 0.027 |
| PDE7B | **3.92** | 0.083 |
| SAT1 | **3.92** | 0.043 |
| SAT1 | **3.92** | 0.146 |
| SOX4 | **3.92** | 0.004 |
| CLU | **3.92** | 0.032 |
| IGFBP5 | **4.06** | 0.045 |
| SEMA6D | **4.06** | 0.108 |
| SPTSSA | **4.06** | 0.055 |
| CDO1 | **4.06** | 0.018 |
| GLCCI1 | **4.06** | 0.103 |
| IGF1 | **4.06** | 0.044 |
| P4HA3 | **4.06** | 0.124 |
| PLXNA4 | **4.06** | 0.163 |
| ENTPD1 | **4.19** | 0.124 |
| AFF3 | **4.19** | 0.114 |
| ATP8B1 | **4.19** | 0.107 |
| MME | **4.19** | 0.011 |
| GNAL | **4.19** | 0.056 |
| NAP1L1 | **4.19** | 0.068 |
| PRL | **4.19** | 0.025 |
| SCARA5 | **4.19** | 0.141 |
| SPON1 | **4.19** | 0.036 |
| BTBD11 | **4.19** | 0.123 |
| PLXDC2 | **4.33** | 0.199 |
| PPP2R1B | **4.33** | 0.086 |
| CHI3L2 | **4.33** | 0.051 |
| PPP1R14A | **4.33** | 0.114 |
| LIFR | **4.33** | 0.101 |
| RNF144A | **4.33** | 0.015 |
| TF | **4.33** | 0.011 |
| EDNRB | **4.48** | 0.018 |
| SESN3 | **4.48** | 0.098 |
| CHMP1B | **4.48** | 0.068 |
| MASP1 | **4.48** | 0.159 |
| TGFB3 | **4.48** | 0.040 |
| HDAC4 | **4.63** | 0.124 |
| LBP | **4.63** | 0.045 |
| PI15 | **4.63** | 0.143 |
| PLAU | **4.63** | 0.025 |
| SLC5A3 | **4.63** | 0.052 |
| STAT4 | **4.63** | 0.030 |
| STEAP2 | **4.63** | 0.104 |
| TBX3 | **4.63** | 0.079 |
| LIFR | **4.79** | 0.100 |
| STMN2 | **4.79** | 0.008 |
| GFPT2 | **4.79** | 0.021 |
| PLD1 | **4.79** | 0.110 |
| RERG | **4.79** | 0.121 |
| RORB | **4.79** | 0.153 |
| TM4SF1 | **4.79** | 0.035 |
| BEND5 | **4.95** | 0.078 |
| NAP1L1 | **4.95** | 0.068 |
| SFRP4 | **4.95** | 0.017 |
| TNFRSF21 | **4.95** | 0.070 |
| STEAP4 | **4.95** | 0.001 |
| FLRT3 | **4.95** | 0.074 |
| PGM2L1 | **4.95** | 0.197 |
| IGFBP5 | **5.12** | 0.011 |
| TNFAIP6 | **5.12** | 0.029 |
| WNT4 | **5.12** | 0.032 |
| FZD1 | **5.29** | 0.186 |
| CCL2 | **5.29** | 0.065 |
| HLA-DPA1 | **5.29** | 0.046 |
| STAMBPL1 | **5.29** | 0.119 |
| FOXQ1 | **5.47** | 0.115 |
| LGR4 | **5.47** | 0.069 |
| HS6ST1 | **5.47** | 0.098 |
| SLC1A3 | **5.47** | 0.007 |
| SIK2 | **5.47** | 0.089 |
| SULF2 | **5.47** | 0.093 |
| KANK4 | **5.47** | 0.132 |
| PTGS1 | **5.66** | 0.209 |
| FLRT3 | **5.66** | 0.087 |
| GALNT15 | **5.66** | 0.210 |
| PDGFRL | **5.66** | 0.022 |
| SOX4 | **5.85** | 0.003 |
| ANGPTL2 | **5.85** | 0.050 |
| SPTSSA | **5.85** | 0.046 |
| FLRT2 | **5.85** | 0.018 |
| DDIT4L | **6.05** | 0.121 |
| MAST4 | **6.25** | 0.102 |
| KCNK3 | **6.25** | 0.170 |
| GLT8D2 | **6.25** | 0.114 |
| HMCN1 | **6.25** | 0.184 |
| NIM1K | **6.25** | 0.153 |
| MME | **6.25** | 0.011 |
| SAT1 | **6.25** | 0.011 |
| FAM162A | **6.25** | 0.181 |
| PLXDC2 | **6.47** | 0.115 |
| ADAM12 | **6.69** | 0.112 |
| CSGALNACT1 | **6.69** | 0.072 |
| MAP7 | **6.69** | 0.007 |
| PCSK1 | **6.69** | 0.027 |
| HDAC4 | **6.91** | 0.018 |
| MRPS6 | **6.91** | 0.094 |
| TGFBI | **6.91** | 0.004 |
| BNC2 | **7.15** | 0.200 |
| CNTNAP2 | **7.15** | 0.075 |
| CDKN1C | **7.39** | 0.054 |
| PDE4D | **7.64** | 0.128 |
| SNCAIP | \| **7.64** \| \| --- \| | 0.077 |
| CXCL12 | **7.90** | 0.040 |
| ADAMTS6 | **7.90** | 0.190 |
| IGF1 | **8.17** | 0.038 |
| RORA | **8.17** | 0.042 |
| BCL3 | **8.44** | 0.021 |
| KIAA1217 | **8.44** | 0.000 |
| PHLDA1 | **8.44** | 0.103 |
| COLEC12 | **8.73** | 0.085 |
| EFHC2 | **8.73** | 0.084 |
| PITPNC1 | **8.73** | 0.073 |
| MGP | **9.03** | 0.006 |
| PTP4A1 | **9.33** | 0.002 |
| RNF175 | **9.33** | 0.074 |
| AIM1 | **9.33** | 0.046 |
| SCG2 | **9.33** | 0.015 |
| ID4 | **9.65** | 0.033 |
| MMP2 | **9.65** | 0.002 |
| PRDM1 | **9.65** | 0.131 |
| IGF1 | **9.97** | 0.038 |
| KCNE4 | **10.31** | 0.087 |
| GNG2 | **10.66** | 0.096 |
| PPARGC1A | **10.66** | 0.074 |
| ST3GAL5 | **10.66** | 0.008 |
| CNTNAP2 | **11.02** | 0.075 |
| TIFA | **11.02** | 0.106 |
| APOE | **11.40** | 0.010 |
| BNC2 | **11.40** | 0.149 |
| APELA | **11.40** | 0.002 |
| SMIM3 | **11.40** | 0.089 |
| ABL1 | **11.78** | 0.006 |
| PLSCR1 | **12.60** | 0.007 |
| SPON1 | **12.60** | 0.056 |
| ADAM12 | **12.60** | 0.055 |
| LOC440895 | **12.60** | 0.131 |
| CHRDL1 | **13.02** | 0.041 |
| STC1 | **13.02** | 0.151 |
| TF | **13.02** | 0.058 |
| ARHGAP28 | **13.02** | 0.121 |
| TANC2 | **13.02** | 0.096 |
| SLIT3 | **13.46** | 0.013 |
| GEM | **13.46** | 0.019 |
| RORA | **13.46** | 0.111 |
| CRISPLD2 | **14.39** | 0.085 |
| PCDHB2 | **14.39** | 0.155 |
| PIK3R3 | **14.39** | 0.007 |
| SFRP4 | **14.39** | 0.017 |
| TNFAIP6 | **14.88** | 0.010 |
| WISP1 | **15.38** | 0.172 |
| PAPPA | **15.91** | 0.096 |
| RAB20 | **15.91** | 0.077 |
| RBP4 | **15.91** | 0.073 |
| WWC1 | **15.91** | 0.051 |
| FKBP7 | **16.44** | 0.154 |
| GNAL | **16.44** | 0.031 |
| ANGPTL2 | **17.58** | 0.050 |
| FAM167A | **18.17** | 0.109 |
| GPR155 | **18.17** | 0.154 |
| IGFBP5 | **18.17** | 0.001 |
| SCARA5 | **18.17** | 0.178 |
| SPAG4 | **18.17** | 0.080 |
| SPON1 | **18.17** | 0.057 |
| CLU | **18.79** | 0.086 |
| PGM2L1 | **18.79** | 0.138 |
| LINC00473 | **19.43** | 0.049 |
| STEAP4 | **19.43** | 0.105 |
| KIAA1217 | **19.43** | 0.159 |
| SAT1 | **19.43** | 0.056 |
| EPHB1 | **20.77** | 0.149 |
| GMNN | **20.77** | 0.069 |
| KALRN | **20.77** | 0.120 |
| KCNJ8 | **20.77** | 0.023 |
| DUSP4 | **21.47** | 0.015 |
| STEAP1 | **21.47** | 0.026 |
| PCOLCE2 | **22.20** | 0.074 |
| SLC16A6 | **22.95** | 0.152 |
| ANTXR1 | **22.95** | 0.080 |
| CXCL12 | **22.95** | 0.012 |
| SPON1 | **22.95** | 0.036 |
| VASH2 | **25.36** | 0.171 |
| ALG9 | **26.22** | 0.127 |
| MID1 | **27.11** | 0.012 |
| FAM212B | **28.03** | 0.228 |
| MMP16 | **28.03** | 0.092 |
| MID1 | **28.03** | 0.011 |
| PDE4D | **28.98** | 0.019 |
| GOLM1 | **30.98** | 0.068 |
| PITPNC1 | **34.24** | 0.207 |
| PLA2G4A | **37.84** | 0.042 |
| SLC22A23 | **40.45** | 0.088 |
| INHBE | **40.45** | 0.043 |
| GALNT15 | **41.82** | 0.186 |
| FAM26F | **46.22** | 0.137 |
| LAMA4 | **47.78** | 0.006 |
| CCL8 | **47.78** | 0.110 |
| PCDHB10 | **49.40** | 0.092 |
| CXCL1 | **49.40** | 0.019 |
| RERG | **54.60** | 0.293 |
| BHLHE40 | **56.45** | 0.003 |
| DTWD1 | **56.45** | 0.166 |
| ANKRD29 | **64.50** | 0.195 |
| CXCL13 | **68.95** | 0.023 |
| KIF26B | **71.28** | 0.203 |
| SOBP | **73.70** | 0.071 |
| TNFSF11 | **75.78** | 0.214 |
| PDZRN3 | **78.78** | 0.047 |
| ENOX1 | **78.78** | 0.077 |
| PLXDC2 | **93.07** | 0.114 |
| CXCL6 | **93.07** | 0.031 |
| TMEM155 | **93.07** | 0.145 |
| FZD1 | **106.34** | 0.019 |
| PGF | **125.63** | 0.039 |
| ADAMTS5 | **138.84** | 0.135 |
| MCC | **158.64** | 0.107 |
| ACKR3 | **169.58** | 0.050 |
| ADAM12 | **200.34** | 0.007 |
| SERINC5 | **200.34** | 0.047 |
| FAM198B | **252.99** | 0.089 |
| FLRT2 | **309.00** | 0.018 |
| MARCKSL1 | **330.30** | 0.002 |
| HEPH | **353.07** | 0.014 |
| PDE7B | **665.14** | 0.144 |
| ITPRIP | **959.74** | 0.268 |
| RAB27B | **1,060.68** | 0.124 |
|  |  |  |

**S1 table. Under- and over-expressed genes were obtained merging the overlapping genes coming from three approaches:** 1) paired pair-wise comparisons using the Affymetrix GCOS comparison algorithm, 2) using dChip Compare Sample procedure, 3) paired t-test using Partek GS®. FC, fold change. T test, paired t-test applied comparing LV-Gsα^R201C^ to mock treated hBMSCs.

**S1 Fig. In silico analysis.** (A) Flow chart of the different steps applied for the functional and statistical analysis of array data. (B) Functional pathways were evaluated in the Gsα^R201C^ data set by Ingeuity Pathwaay Analysis (IPA). Significant pathways were defined by assessing the number of molecules mapping to the pathway and by Fisher’s exact test calculated p-value. Top ten scoring pathways are showed in the histogram.

**S2 Fig. qPCR analysis.** (A)  qPCR analysis on cDNA extracted from LV-Gsα^R201C^ and mock treated hBMSCs, for the different indicated genes. Data are obtained from tripilicate measurements of independent biological duplicates shown as the difference of LV-Gsα^R201C^ fold change respect to mock treated samples, each dot represent an individual sample. *P* values were calculated with Student’s *t* test (**p* < .05, ** *p* < .01 ****p* < .001).
