# Supplementary figures and images for: Changes in gene expression in human skeletal stem cells transduced with constitutively active Gsα correlates with hallmark histopathological changes seen in fibrous dysplastic bone
Source: PLoS One. 2020 Jan 30;15(1):e0227279. doi: 10.1371/journal.pone.0227279 (PMC6991960; doi:10.1371/journal.pone.0227279)

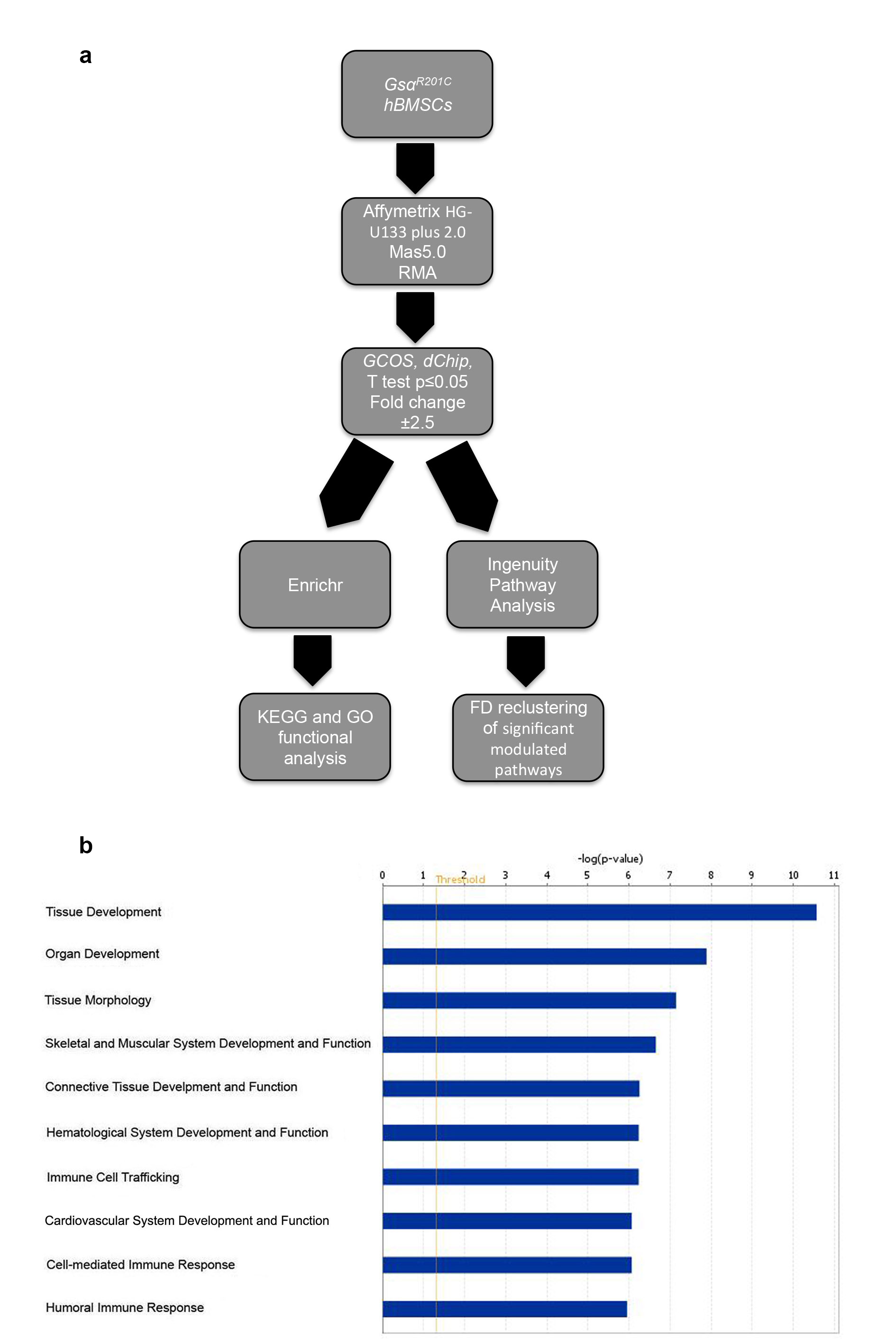

Supplement: S1 Fig — (A) Flow chart of the different steps applied for the functional and statistical analysis of array data. (B) Functional pathways were evaluated in the GsαR201C data set by Ingeuity Pathwaay Analysis (IPA). Significant pathways were defined by assessing the number of molecules mapping to the pathway and by Fisher’s exact test calculated p-value. Top ten scoring pathways are showed in the histogram. (TIF) [file pone.0227279.s002.tif]

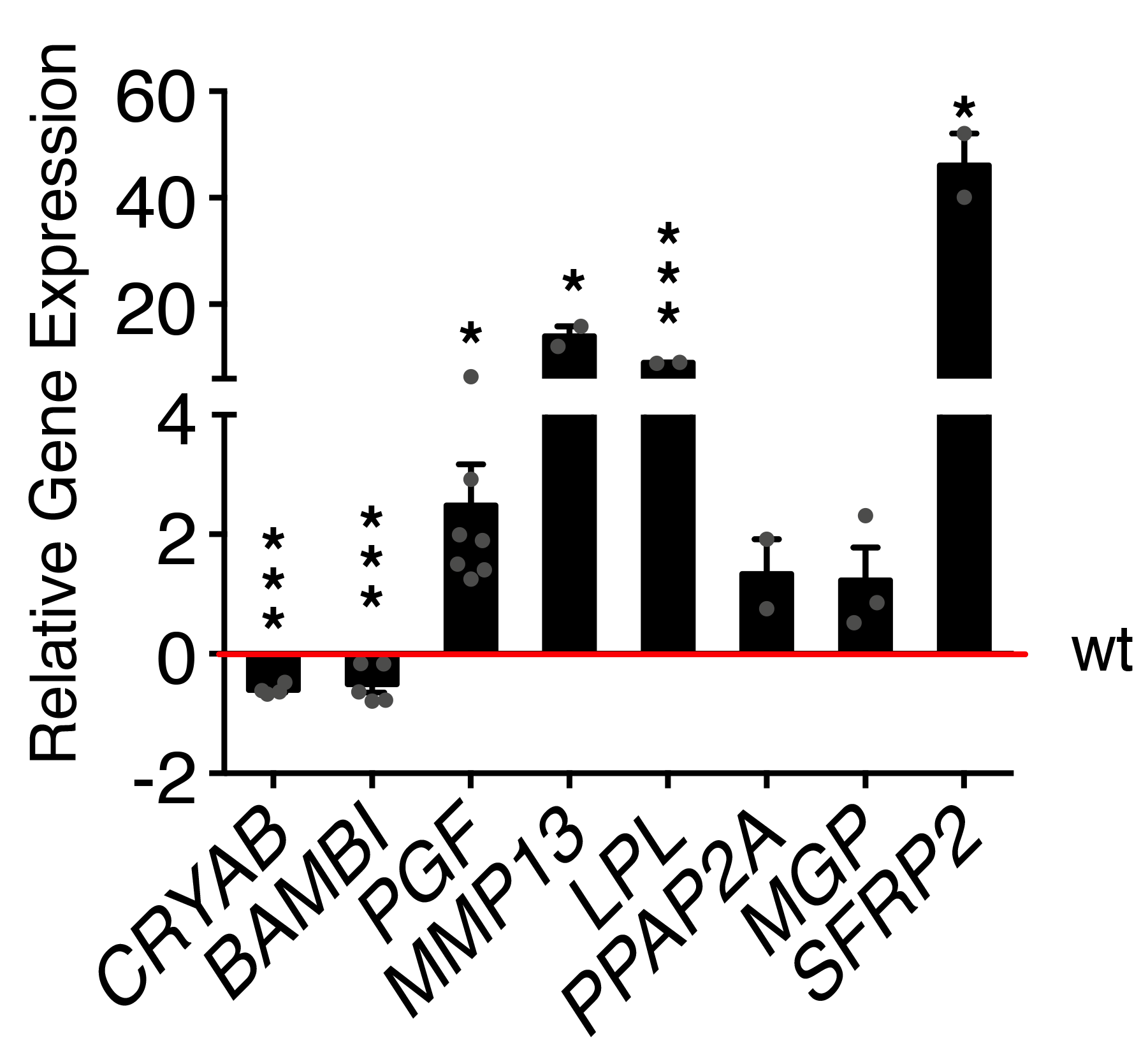

Supplement: S2 Fig — (A) qPCR analysis on cDNA extracted from LV-GsαR201C and mock treated hBMSCs, for the different indicated genes. Data are obtained from tripilicate measurements of independent biological duplicates shown as the difference of LV-Gsα\R201C fold change respect to mock treated samples, each dot represent an individual sample. P values were calculated with Student’s t test (*p < .05, ** p < .01 ***p < .001). (TIFF) [file pone.0227279.s003.tiff]
