# Supplementary material for: Changes in gene expression in human skeletal stem cells transduced with constitutively active Gsα correlates with hallmark histopathological changes seen in fibrous dysplastic bone
Source: PLoS One. 2020 Jan 30;15(1):e0227279. doi: 10.1371/journal.pone.0227279 (PMC6991960; doi:10.1371/journal.pone.0227279)
Supplement: Attachment — Submitted filename: cover rebuttal gnas array 261119.doc [file pone.0227279.s004.doc]

November 26, 2019

**PONE-D-19-26235**

Dear Editor,

We enclose the revised version of the manuscript “Changes in gene expression in human skeletal stem cells transduced with constitutively active Gsα correlates with hallmark histopathological changes seen in fibrous dysplastic bone” by Domenico Raimondo and coworkers.

We hope that you will find this revised version suited for publication in PLOS One.

Thank you for your consideration,

Isabella Saggio

[isabella.saggio@uniroma1.it](mailto:isabella.saggio@uniroma1.it),

**PONE-D-19-26235 - REBUTTAL LETTER**

*Reviewer #1:*

This manuscript describes gene expression changes (assessed by microarrays) in human bone marrow stromal cells in response to introduction of the FD-associated Gs,alpha R201C mutation. This paper is well-written and contains a systematic and careful discussion about the complex nature of bone disease in FD. THis new dataset will be of some value to researchers studying FD and other cAMP-regulated bone conditions. Overall, these microarray data are clearly described and robust.

**We thank for the favourable comments.**

Minor comments:
1. In Figure 1A, the three samples transduced with the R201C mutant are all listed as D02R. Is this correct? Or should this instead state D01R, D02R, and D03R? Ideally the authors would perform this analysis using three separate BMSC donors.

**The analysis was indeed performed on three donors and the Figure was corrected.**

2. It would be useful to show an immunoblot demonstrating expression levels of the R201C mutant compared to endogenous Gs,alpha protein levels.

**This experiment was performed and added as Figure 1A.**

3. The authors could compare/contrast the genes regulated by Gs,alpha R201C expression and genes whose expression is regulated by continuous hyperparathyroidism (PMID 17690103).

**This comparison was performed and added as Figure 7C.**

*Reviewer #2:*

The manuscript by Raimondo et al., reports transcriptome analysis of human bone marrow stromal cells constitutively expressing a mutated dominant form of the GNAS gene coding for a G-protein coupled receptor. The mutation is a substitution of Arginine to Cysteine at position 201 of the alpha subunit and causes fibrous dysplasia of bone. The cells are transduced with a lentiviral vector carrying the GsαR201C mutation. Previous work by the group has documented that GsαR201C transduced cells manifest the diseased phenotype in vitro and after ectopic in vivo transplantation into mice. In this report the authors concentrate on the expression profile of transduced cells with the aim to find early perturbations in gene expression that may provide potential targets for development of therapeutic strategies. For this, 3 different human samples isolated from healthy individuals were transduced with the constitutive active GsαR201C or with control vectors and expression profile analysis was performed after 15 days. The bioinformatics analysis of the data was directed towards the identification of pathways that may relate to disease phenotype. Interestingly genes belonging to the Wnt pathway as well as genes related to osteoblastogenesis were dysregulated in GsαR201C transduced cells and provide a link to the bone malformation situation observed in fibrous dysplasia (FD) patients. Similarly, an altered adipogenic program is related the block of adipogenesis in FD patients. Interestingly, comparison of the expression profile in this manuscript to published data from FD patients revealed common sets of dysregulated genes. Despite that the expression of GsαR201C is regulated from an exogenous promoter and not from the endogenous locus, the transcriptomic data are informative and can contribute to a better understanding of the molecular pathways involved in FD disease.

Minor comments

1. Please describe better the culture requirements for hBMSCs in the “Material and Methods” part. A far as I know, dexamethasone and ascorbic acid are used for osteogenic differentiation and not for maintaining hBMSCs. Further, you should mention why you culture 293T cells (virus production?).

**293T cells were used only for virus production and this was indicated in the Materials and Methods section. The protocol for hBMSCs was corrected, there was a mistake in the text and in reference positioning.**

2. KLF2 is Krüppel-like Factor 2 and NOT Krüpple

**This was corrected in the text.**
